# The Book World of Medicine and Science

**Published:** 1900-12-29

**Authors:** 


### The Book World of Medicine and Science.

Golden Rules of Skin Practice. By David Walsh, M.D.Edin. Golden Rules Series. (Bristol: John Wright and Co. 1900. Price 1s.)

This is another of the useful little pocket-books which form the so-called Golden Rules Series. There is no doubt that it is very well done, for the little pages of this tiny volume contain a very large amount of information on all sorts of subjects connected with the treatment of skin diseases, many of them points which require to be constantly borne in mind if one would succeed in practice. The somewhat irritating artificiality in the arrangement of the paragraphs which is a marked feature of this series is, we suppose, due to the requirements of the publishers and the exigencies of "golden rules." To some minds it may perhaps add force to a sentence to head it with such words as "Remember" or "Bear in mind" or "Carefully distinguish" or "Never neglect," and to print these words in heavy type, but to ordinary mortals the constant reiteration of such personal appeals appears an annoying and rather meaningless formulary. Still the stuff within the sentences is mostly good, and the "tips" are many, so that we have no doubt that the little book will prove both popular and useful.

The Essentials of Practical Bacteriology: an Elementary Laboratory Book for Students and Practitioners. By H. J. Curtis, B.Sc. and M.D. (Lond.), F.R.C.S. (Longmans, Green, & Co. 1900. Price 9s.)

The strong point of this book is its thoroughness, the descriptions of the various proceedings being so well thought out and so carefully arranged that if ever we should feel inclined to say that book teaching could take the place of practical instruction, we should say it of this work. And perhaps in this will be found one of the utilities of such a manual. Bacteriology has of late become a matter of immense importance to practitioners, many of whom, although they have time enough and opportunities enough to become fairly expert in such of its details as are of most use from a clinical point of view, cannot possibly leave their practices to obtain instruction in the technique of the art. To these this book will prove invaluable. No doubt it is not to them that its pages are specially addressed, it being in reality a complete handbook to laboratory work, particularly designed for the Public Health Diploma; but so accurate and thorough are the descriptions given that we can hardly doubt that any practitioner who chose to follow its directions could make himself an expert bacteriologist—at any rate, after a very short practical course, just to give him the key as to what to look for. Part I. is devoted to the manufacture of nutrient media and to general technique, and in it we are introduced to all the tricks and dodges of the laboratory, the apparatus, the solutions, the making of preparations, the use of stains, the methods of cultivating micro-organisms, and the various ways of recording observations. Part II. is given up to a systematic study of the various micro-organisms. The non-pathogenic organisms are first described, including the bacteria and bacilli, the yeasts, the putrefactive organisms, the moulds, and—by what appears to us an oversight in the division of the subjects—the moulds of ringworm and of favus. Then we come to the investigation of the pathogenic organisms, properly so called, and to this the major part of the book is devoted. In this portion of the work the author has very properly included what some would exclude from bacteriology pure and simple, namely, the malaria parasite the amœbæ of dysentery, and the parasite of cancer; then comes a chapter on the bacteriological examination of air, water, soil, and milk; and finally another on the methods employed in testing the efficacy of various antiseptics and disinfectants. Finally there are a series of appendices which add to the value of the work as a book of reference. The whole book is well got up, and the illustrations are numerous and very good. As we have said, Dr. Curtis has here written a handbook primarily intended for those going through the course of study generally required for the Diploma in Public Health, and by the careful directions which he has given and by his discriminating remarks in regard to the distinguishing points by which the various micro-organisms may be differentiated from one another he has done much to lighten their labours and to smooth their path. But he has done more, for he has opened a way by which many a practitioner more or less cut off, as so many are, from the society of scientific men may, if he chooses, make for himself that useful thing a hobby, and one, moreover, in the riding of which he need never feel that he is entirely wasting time; for, although bacteriology lies sufficiently out of the ordinary track of the daily round to make its cultivation a pleasure and a change, it must always remain the handmaid, as it has lately been the guiding spirit, of scientific medicine.

### NOTES ON BOOKS.

Among the various reminders of Christmas which fall into the hands of practitioners at this time of the year are the visiting lists and pocket diaries with which our ever-ready friends, the wholesale chemists so lavishly provide us. "Ephemeris Pharmacologica" (Oppenheimer, Son and Co.) is a prettily-bound, flat-lying pocket-book, containing a great deal of information, not merely about drugs, but concerning the hundred and one things that a medical man wants to know on the spur of the moment. The printed pages are followed by a very nicely arranged visiting list, with the usual spaces for obstetric and other engagements, and for memoranda of various kinds. The portion of this pocket-book which more especially bears the heading "Ephemeris Pharmacologica" consists of brief notes on the uses and properties of new drugs, &c.; and very well it is done. Of course the true inwardness of the whole thing is to bring before the notice of medical men the palatinoids and other special preparations with which the name of the firm of Oppenheimer is specially associated; but all the same it contains much information, and the palatinoid side of the question is not made unduly prominent.

"Wellcome's Medical Diary and Visiting List, 1901" (Burroughs, Wellcome, and Co.) comes to the recipients packed up in quite a lavish sample box of the various productions of the firm by which it is issued, especially of the "tabloids," for the introduction of which into medical practice the firm of Burroughs and Wellcome is probably best known throughout the world. As for the book itself it is very full of matter, but is rather too thick to carry comfortably in the pocket, although admirable for the doctor's bag. The visiting list is well arranged, and the information contained in the printed portion can only be described by the word "immense."

"Scott's Emulsion Pocket Diary and Note Book" (Scott and Bowne) is a neat little diary for the pocket, nicely bound and free from all trace of printed padding except such as is common to most diaries, and containing an accident coupon for £100. Then there is the diary issued by the Maltine Company, and half-a-dozen others, while of Calendars there is no end, all of which crowd the doctor's table and ultimately reach various unexpected destinations. We cannot say we like the system of scattering these half-presents, half-advertisements broadcast throughout the doctors of the land, but at least there is safety in numbers. No one can say that a medical man is influenced by the acceptance of a diary when he has half-a-dozen of them in his desk to choose from.

